# Progression of Visual Pathway Degeneration in Primary Open-Angle Glaucoma: A Longitudinal Study

**DOI:** 10.3389/fnhum.2021.630898

**Published:** 2021-03-29

**Authors:** Shereif Haykal, Nomdo M. Jansonius, Frans W. Cornelissen

**Affiliations:** ^1^Laboratory for Experimental Ophthalmology, University Medical Center Groningen, University of Groningen, Groningen, Netherlands; ^2^Department of Ophthalmology, University Medical Center Groningen, University of Groningen, Groningen, Netherlands

**Keywords:** glaucoma, white matter, diffusion MRI, fixel-based analysis, longitudinal

## Abstract

**Background:** Primary open-angle glaucoma (POAG) patients exhibit widespread white matter (WM) degeneration throughout their visual pathways. Whether this degeneration starts at the pre- or post-geniculate pathways remains unclear. In this longitudinal study, we assess the progression of WM degeneration exhibited by the pre-geniculate optic tracts (OTs) and the post-geniculate optic radiations (ORs) of POAG patients over time, aiming to determine the source and pattern of spread of this degeneration.

**Methods:** Diffusion-weighted MRI scans were acquired for 12 POAG patients and 14 controls at two time-points 5.4 ± 2.1 years apart. Fiber density (FD), an estimate of WM axonal density, was computed for the OTs and ORs of all participants in an unbiased longitudinal population template space. First, FD was compared between POAG patients and the controls at time-point 1 (TP1) and time-point 2 (TP2) independently. Secondly, repeated measures analysis was performed for FD change in POAG patients between the two time-points. Finally, we compared the rate of FD change over time between the two groups.

**Results:** Compared to the controls, POAG patients exhibited significantly lower FD in the left OT at TP1 and in both OTs and the left OR at TP2. POAG patients showed a significant loss of FD between the time-points in the right OT and both ORs, while the left OR showed a significantly higher rate of FD loss in POAG patients compared to the controls.

**Conclusions:** We find longitudinal progression of neurodegenerative WM changes in both the pre- and post-geniculate visual pathways of POAG patients. The pattern of changes suggests that glaucomatous WM degeneration starts at the pre-geniculate pathways and then spreads to the post-geniculate pathways. Furthermore, we find evidence that the trans-synaptic spread of glaucomatous degeneration to the post-geniculate pathways is a prolonged process which continues in the absence of detectable pre-geniculate degenerative progression. This suggests the presence of a time window for salvaging intact post-geniculate pathways, which could prove to be a viable therapeutic target in the future.

## Introduction

Primary open-angle glaucoma (POAG) is a degenerative optic neuropathy and a major cause of irreversible blindness worldwide (Tham et al., 2014). POAG is characterized by the death of retinal ganglion cells (RGCs) and progressive visual field loss (Weinreb and Khaw, 2004). While an increase in intraocular pressure is recognized as a major risk factor, the underlying pathophysiology of POAG remains unclear (Weinreb et al., 2014).

Over the past decade, cross-sectional MRI studies of POAG patients have found evidence of structural degeneration along the entire length of their visual pathways (Garaci et al., 2009; Engelhorn et al., 2011; Bolacchi et al., 2012; Nucci et al., 2012; Zikou et al., 2012; Chen et al., 2013; El-Rafei et al., 2013; Lu et al., 2013; Michelson et al., 2013; Murai et al., 2013; Wang et al., 2013; Frezzotti et al., 2014, 2016; Kaushik et al., 2014; Omodaka et al., 2014; Sidek et al., 2014; Tellouck et al., 2016; Zhou et al., 2017; Song et al., 2018; Li et al., 2019; You et al., 2019). Degeneration was reported in both pre-geniculate pathways, including the optic nerves and optic tracts (OTs), and post-geniculate pathways, comprising the optic radiations (ORs) and visual cortex. The cause of the reported degeneration in post-geniculate pathways is still a matter of debate. The spread of glaucomatous degeneration from pre- to post-geniculate pathways through anterograde trans-synaptic degeneration is the most widely accepted explanation (Calkins and Horner, 2012). However, some have suggested that glaucomatous degeneration originates in the post-geniculate pathways and then reaches the pre-geniculate pathways through retrograde trans-synaptic degeneration, eventually producing the characteristic RGCs death and vision loss found in POAG (Davis et al., 2016; Lawlor et al., 2017), although this is not a mainstream opinion. If retrograde trans-synaptic degeneration is in fact responsible for the degenerative changes found in the pre-geniculate pathways, and hence the retina, POAG could be considered as a primarily degenerative disease of the brain rather than the eyes. Additionally, some MRI studies have reported degenerative changes beyond vision-related areas of the brain, further suggesting the presence of an independent brain component in POAG (Frezzotti et al., 2014, 2016).

Using a novel method for analyzing diffusion-weighted MRI (DWI) known as fixel-based analysis (FBA; Raffelt et al., 2017), we have recently characterized degenerative WM changes of the visual pathways in POAG in terms of microstructural axonal loss and macrostructural atrophy (Haykal et al., 2019). In contrast to conventional methods of DWI analysis, FBA produces biologically interpretable measures of white matter (WM) structural degeneration. These measures are: fiber density (FD), fiber-bundle cross section (FC), and fiber density and bundle cross section (FDC). FD is an estimate of axonal density in a WM fiber bundle, reflecting degeneration on a microstructural scale. FC, on the other hand, is a morphological measure of changes in cross-sectional area experienced by a WM fiber bundle, reflecting degeneration on a more macrostructural scale. FDC is a combined measure of both FD and FC, reflecting the overall information carrying capacity of a WM fiber bundle. Using FBA, we found evidence of microstructural axonal loss in the OTs and ORs of POAG patients and evidence of macrostructural atrophy in their OTs only. Animal studies have indicated that axonal loss precedes atrophic changes in glaucomatous WM degeneration (Ito et al., 2009). Therefore, we proposed that our findings could be evidence that the OTs were exhibiting signs of later stages of degeneration compared to the ORs. In turn, this would imply that glaucomatous degeneration of the OTs precedes that of the ORs, hence implicating anterograde trans-synaptic degeneration. However, as our study was cross-sectional in nature, this interpretation remained strictly speculative. In fact, to date, all MRI studies of WM degeneration in POAG have been cross-sectional, limiting their ability to identify the source and pattern of progression of this degeneration over time.

To address this issue, we re-invited participants of two of our previous DWI studies of POAG (Hernowo, 2012; Hanekamp, 2017) to assess the progression of WM degeneration exhibited by their visual pathways since the initial studies were undertaken. We used FBA to investigate degenerative progression in the pre-geniculate OTs and the post-geniculate ORs of the POAG patients compared to the controls. By doing so, we aimed to determine the source and pattern of spread of visual pathway WM degeneration in POAG.

## Materials and Methods

### Ethical Approval

This study adhered to the tenets of the Declaration of Helsinki and was approved by the ethics board of the University Medical Center Groningen (Approval number: 2017/232). A written informed consent was provided by all participants.

### Participants

During the periods of April 2008 to December 2009 and May 2013 to June 2014, 27 POAG patients and 27 controls participated in two DWI studies of WM degeneration in POAG (Hernowo, 2012; Hanekamp, 2017). All surviving participants (24 POAG patients and 27 controls) were invited to participate in the current retrospective follow-up investigation, of which 14 POAG patients and 17 controls agreed to participate again. The inclusion criteria for the POAG group were: having participated in one of the two previous DWI studies and being diagnosed with POAG. For POAG, reproducible visual field loss had to be present in at least one eye. The visual field loss had to be compatible with glaucoma, accompanied by glaucomatous optic neuropathy (defined as a vertical cup-to-disc ratio above 0.7 or notching), and without any other explanation. Open angles on gonioscopy were required as well as absence of signs of pigment dispersion, pseudoexfoliation, or secondary causes of glaucomatous optic neuropathy. The inclusion criteria for controls were: having participated in one of the two previous DWI studies, having no visual field defects, having a decimal visual acuity score of 0.8 or higher, and having an intraocular pressure (IOP) of ≤21 mm Hg. General exclusion criteria for both groups were: development of any ophthalmic, neurologic or psychiatric disorders since the initial studies were undertaken, having any contraindication to being inside an MRI scanner, and the detection of apparent lesions or abnormalities in the acquired MRI scans. Two POAG patients were excluded, both for having MRI-incompatible cardiac implantations. Three controls were excluded, one for experiencing a transient ischemic attack since participating in the initial studies, one for having a possible metal shrapnel in an eye, and one for having an IOP >21 mmHg during assessment for recruitment in the current follow-up. In total, 12 POAG patients and 14 controls were included in this longitudinal study (Table 1). Follow-up data was acquired during the period of October 2017 to February 2018. The mean time interval between the initial and the follow-up visits was 6.1 ± 2.4 years for POAG patients and 4.8 ± 1.7 years for controls (*P* = 0.118). From hereon, both initial studies will be referred to as Time-point 1 (TP1) and the follow up investigation will be referred to as Time-point 2 (TP2).

**Table 1:** Group demographics and clinical characteristics at time-point 1 and time-point 2.

	**Time-point 1**			**Time-point 2**		
**Characteristics**	**POAG (*n* = 12)**	**Controls (*n* = 14)**	**Group difference *P***	**POAG (*n* = 12)**	**Controls (*n* = 14)**	**Group difference *P***
Age (y)	60.6(7.1)	62.8(8.0)	0.469	66.5(7.2)	67.4(7.4)	0.979
Male sex	6(50%)	8(57.1%)	0.716	6(50%)	8(57.1%)	0.716
**IOP (mmHg)**						
OD	15.5(4.6)	14.8(3.1)	0.688	12.9(3.3)	12.2(3.2)	0.954
OS	14.7(4.7)	14.5(3.1)	0.906	13.0(4.7)	13.1(3.7)	0.588
Mean	15.1(4.5)	14.7(3.0)	0.792	12.9(3.5)	12.7(3.3)	0.846
**NFI**						
OD	50.5(24.5)^*^	25.5(15.0)^†^	**0.006**	57.9(21.3)	25.9(11.0)	** <0.001**
OS	46.7(27.6)^*^	19.5(9.3)^†^	**0.013**	53.8(27.8)	22.1(8.5)	**0.002**
Mean	48.6(11.6)^*^	22.5(11.0)^†^	** <0.001**	55.9(16.2)	24.0(9.0)	** <0.001**
**VFMD (dB)**						
Worse eye^‡^	−13.3(9.1)^*^	-	-	−14.5(9.7)	-	-
Better eye^‡^	−1.5(1.5)^*^	-	-	−3.3(5.1)	-	-
Mean	−6.9(4.7)^*^	-	-	−8.9(6.1)	-	-

*Values are presented as mean (SD) or number (%). IOP, intraorbital pressure; NFI, nerve fiber indicator; OD, right eye; OS, left eye; VFMD, visual field mean deviation*.

* *Two missing data points*.

† *One missing data point*.

‡ *Worse/better refers to eye with more/less negative VFMD values. Bold values represent statistically significant p-values*.

### Ophthalmic Tests

All included participants underwent the same ophthalmic tests that were performed at TP1. Visual acuity was tested using a Snellen chart with optimal refractive correction for viewing distance. IOP was measured using a Tonoref non-contact tonometer (Nidek, Hiroishi, Japan), which relies on a puff of air to applanate the cornea and estimates the IOP by measuring the force of the air jet at applanation. The RNFL thickness was assessed using laser polarimetry (GDx; Carl Zeiss Meditec, Jena, Germany) and expressed as Nerve Fiber Indicator (NFI). Laser polarimetry involves the projection of a beam of polarized light into the eye and estimating the RNFL thickness based on the phase shift experienced by the beam as it passes through the RNFL. Visual fields in POAG patients were tested using a Humphrey Field Analyzer (HFA; Carl Zeiss Meditec, Jena, Germany) with 30-2 grid and SITA strategy, and the outcome was expressed as visual field mean deviation (VFMD). HFA works by projecting light stimuli of different intensities on a hemisphere covering the visual field being tested, and the patient is asked to press a button once they detect the projected light stimulus. For healthy controls, the visual fields were tested using frequency doubling technology (FDT; Carl Zeiss Meditec, Jena, Germany) C20-1 screening mode to screen for any visual field defects. All test locations had to show no reproducible abnormalities (*P* < 0.01) to be considered intact. FDT uses the frequency doubling phenomenon to test contrast sensitivity in different sections of the visual field and subsequently detect visual field defects. The outcome of the tests and duration of glaucoma for POAG patients are listed in Supplementary Table 1.

### Image Acquisition and Pre-processing

The same MRI scanner and scanning protocol were used to acquire the DWI data for both studies at TP1. To ensure data comparability, the same MRI scanner and scanning protocol used at TP1 were also used in the current follow-up investigation. A Philips Intera 3T MRI scanner (Eindhoven, The Netherlands) was used, with either an 8- or 16-channel head coil, depending on the head coil used for the initial scan of each participant. The scanning parameters were as follows: field of view (FoV) = 240 × 240 × 102 mm, 51 axial slices, voxel size = 1.875 × 1.875 × 2 mm, echo time (TE) = 79 ms, repetition time (TR) = ~5,500 ms, EPI factor = 45, 60 diffusion gradient directions (b= 800 s/mm^2^) acquired in 2 phase-encoding directions (anteroposterior and posteroanterior) with a single b = 0 s/mm^2^ volume in each phase-encoding direction.

The acquired DWI data was then preprocessed, which included denoising to improve the signal-to-noise ratio (Veraart et al., 2016), correction for susceptibility (Andersson et al., 2003), motion and Eddy current induced distortions (Andersson and Sotiropoulos, 2016) in FMRIB's Software Library (FSL v5.011, https://fsl.fmrib.ox.ac.uk/fsl). Finally, bias field inhomogeneity correction (Tustison et al., 2010) and global intensity normalization was performed.

### Fixel-Based Analysis

All FBA steps were performed using MRtrix3 (Tournier et al., 2019) according to the recommended FBA pipeline (Raffelt et al., 2017), unless specified otherwise. First, an average WM response function was derived from the DWI data of all participants (Tournier et al., 2013). Then, the DWI data was upsampled to an isotropic voxel size of 1.3 mm, and a fiber orientation distribution (FOD) map was produced for each participant using the average WM response function.

**Figure 1:**
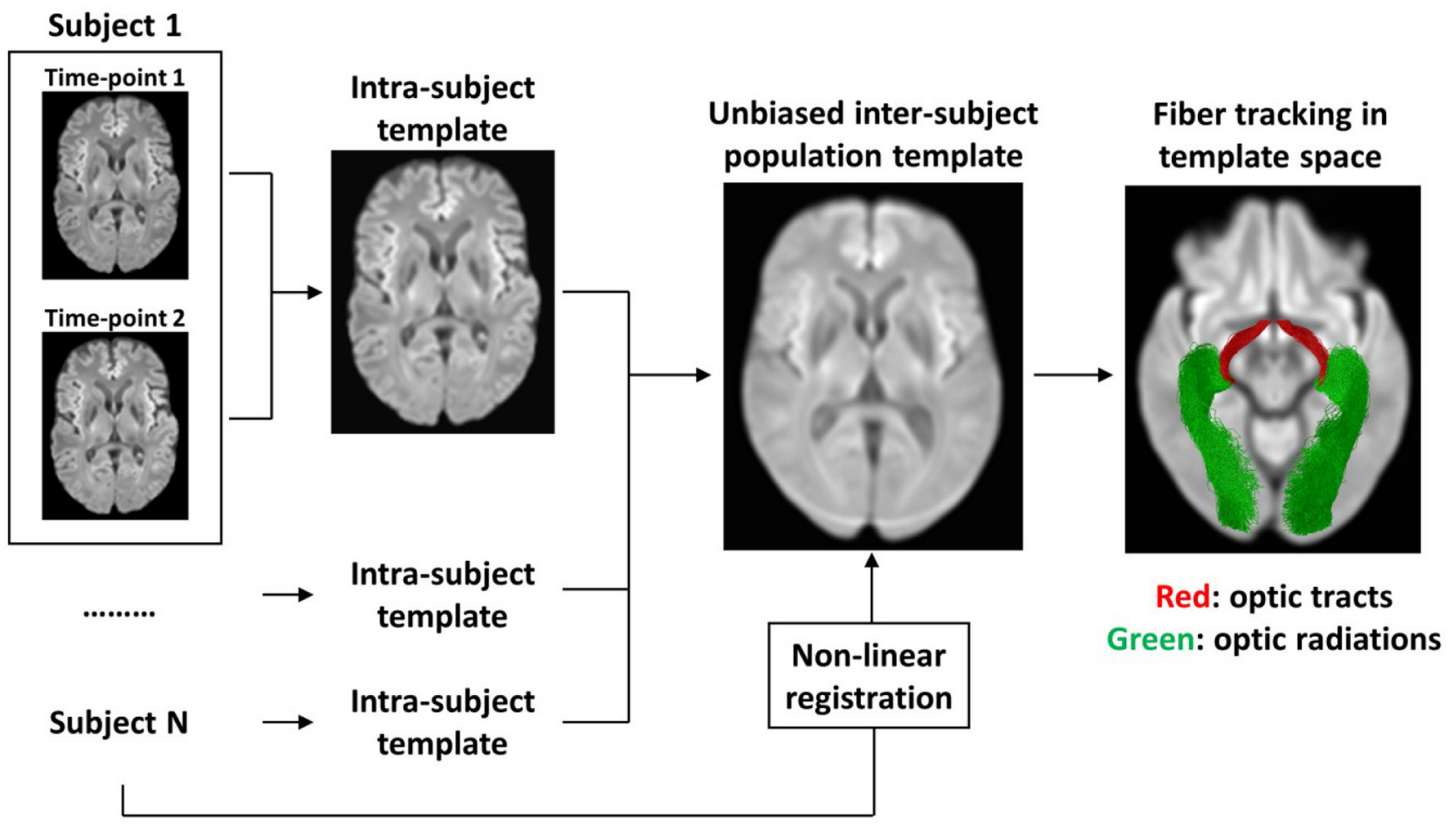
Flow-chart summarizing the main steps for creating an unbiased longitudinal population template. First, an intra-subject template was created from TP1 and TP2 scans of each participant individually. Then, the intra-subject templates of all participants were used to create an unbiased inter-subject population template. To achieve spatial correspondence between all participants in template space, each of the participant's scans were warped from native space to template space using non-linear registration. To specifically analyze the visual pathways, the OTs and the ORs were tracked using probabilistic tractography in template space.

To build an unbiased longitudinal population template, we adapted a method described previously by Genc and colleagues (Genc et al., 2018). Figure 1 outlines the main steps followed. First, the FOD maps of each participant at TP1 and TP2 were co-registered to midway space using rigid body transformation, followed by the production of an intra-subject population template using iterative non-linear registration and averaging of both FOD maps (Raffelt et al., 2011, 2012). The produced intra-subject population templates of all 26 participants were then used to produce an unbiased longitudinal population template as described in the recommended FBA pipeline (Raffelt et al., 2017). Finally, all individual FOD maps were non-linearly registered to the longitudinal population template.

Subsequently, the FOD lobes were segmented to identify the fixels within each voxel. The produced fixels were then reoriented to achieve fixel correspondence with the population template and each fixel was assigned an FD value based on the amplitude of the FOD lobe they represent. Then, FC values were computed for each fixel based on the warps produced during their registration to the population template. Finally, FDC was calculated from the produced FD and FC values.

A whole-brain tractogram was produced using the longitudinal population template to determine local connectivity between fixels and hence allow the use of Connectivity-based Fixel Enhancement (CFE) for statistical inference (Raffelt et al., 2015). A total of 20 million streamlines were produced initially, then they were filtered down to 2 million streamlines using Spherical-deconvolution Informed Filtering of Tractograms (Smith et al., 2013) method to reduce tractography biases. To specifically analyze the visual pathways, the OTs and ORs were tracked in population template space as previously described (Haykal et al., 2019) and then converted to fixel masks to be used as regions-of-interest for FBA (Figure 1).

### Statistical Analysis

#### Demographics and Clinical Characteristics

For group comparisons, independent-samples *t*-test was used for parametric continuous variables of equal variance, Welch's *t*-test was used for parametric continuous variables of unequal variance, Mann-Whitney *U*-test was used for non-parametric continuous variables, and χ^2^ test was used for categorical variables. Statistical significance was reported at *P* < 0.05.

#### Fixel-Based Analyses

Fixel masks produced from the tracked OTs and ORs were used as region-of-interest masks for FBA. All fixel-based analyses used non-parametric permutation testing and CFE for statistical inference (Raffelt et al., 2015). Following 5,000 permutation tests, fixels were assigned a family-wise error (FWE)-corrected *P*-value. Statistical significance was reported at an FWE-corrected *P* < 0.05. To visualize the results, streamline segments corresponding to fixels showing statistically significant outcomes were cropped from the produced population template tractogram and displayed.

We performed both cross-sectional and longitudinal fixel-based analyses of the data. The aim of the cross-sectional analyses was to qualitatively assess the spatial spread of degenerative changes in the POAG group. To do so, independent cross-sectional comparisons between FBA measures of the POAG group and the controls at TP1 and TP2 were performed to identify visual pathways exhibiting significant degeneration at each time-point. Sex, demeaned age at the time of scan, and type of head coil used were included as nuisance covariates.

The aim of the longitudinal analyses was to quantitatively assess the progression of visual pathway degeneration. Two different longitudinal analyses were performed. In the first of these, a repeated measures analysis was used to compare FBA measures of the POAG group at TP1 and TP2. The second longitudinal analysis served to ensure that any degenerative progression detected in the POAG group is not the result of natural age-related WM degeneration over time. To do so, we compared the progression of FBA changes over time between the POAG group and the controls. As the average time interval between the scans was not equal between the two groups (see Participants subsection), a rate of progression of FBA changes was calculated for each participant and used for comparison. For each participant, the rate of progression was calculated by subtracting each FBA metric TP1 image from its corresponding TP2 image, and then dividing the resulting difference image by the time interval in years, as described by Genc and colleagues (Genc et al., 2018). The average rate of progression of all three FBA measures were then compared between the two groups, adding sex, demeaned average age, and type of head coil used as nuisance covariates.

#### Supplementary Confirmatory Analyses

As the POAG group and the controls experienced unequal experimental time intervals on average, the results of the cross-sectional analyses may have been influenced by unequal amounts of natural age-related WM degeneration experienced by both groups. To ensure a qualitative comparability of the spatial spread of degenerative changes between the cross-sectional analyses at both time-points, participants from both groups were matched one-to-one based on approximately equal time intervals. This resulted in a total of nine pairs of time-interval-matched POAG patients and controls (see Supplementary Table 2 for matching details). Following time-interval-matching, the mean time interval was 5.2 ± 2.0 years for both groups. All cross-sectional fixel-wise analyses producing statistically significant results with all participants included were repeated for the time-interval-matched participants to confirm the results.

To further confirm the results of the longitudinal analyses, each FBA metric TP1 image was subtracted from its corresponding TP2 image, and the time interval for each subject was used as a regressor in the analysis. The differences in FBA measures were then compared between the two groups, adding sex, demeaned average age, and type of head coil used as nuisance covariates.

#### Correlation Between FBA Measures and Clinical Tests

To study the correlation between the change in FBA measures and the change in ophthalmic tests results over time in POAG patients, the average FBA measures (FD, FC, and FDC) for each tract (OTs and ORs) were first computed for each patient. Then, the values of the right and left tracts for each computed FBA value were averaged. The results of the ophthalmic tests were also averaged over both eyes for each patient. This was done to allow comparisons between the results of the ophthalmic tests and the FBA measures derived from post-chiasmatic pathways. Missing data points for both NFI and HFA tests were excluded from the analysis. Spearman's correlation analysis was used to investigate the correlation between the differences in the computed measures between the two time-points. Statistical significance was reported at *P* < 0.05.

## Results

### Demographics and Clinical Characteristics

Table 1 summarizes the demographics and clinical characteristics of the POAG and control groups at TP1 and TP2. Participants of both groups did not differ significantly in age or sex at TP1 (*P* = 0.47, *P* = 0.72, respectively) or TP2 (*P* = 0.98, *P* = 0.72, respectively). The POAG group showed a significantly higher mean NFI compared to controls at both time-points (both *P* < 0.001).

### Cross-Sectional Fixel-Based Analyses at Both Time-Points

**Figure 2:**
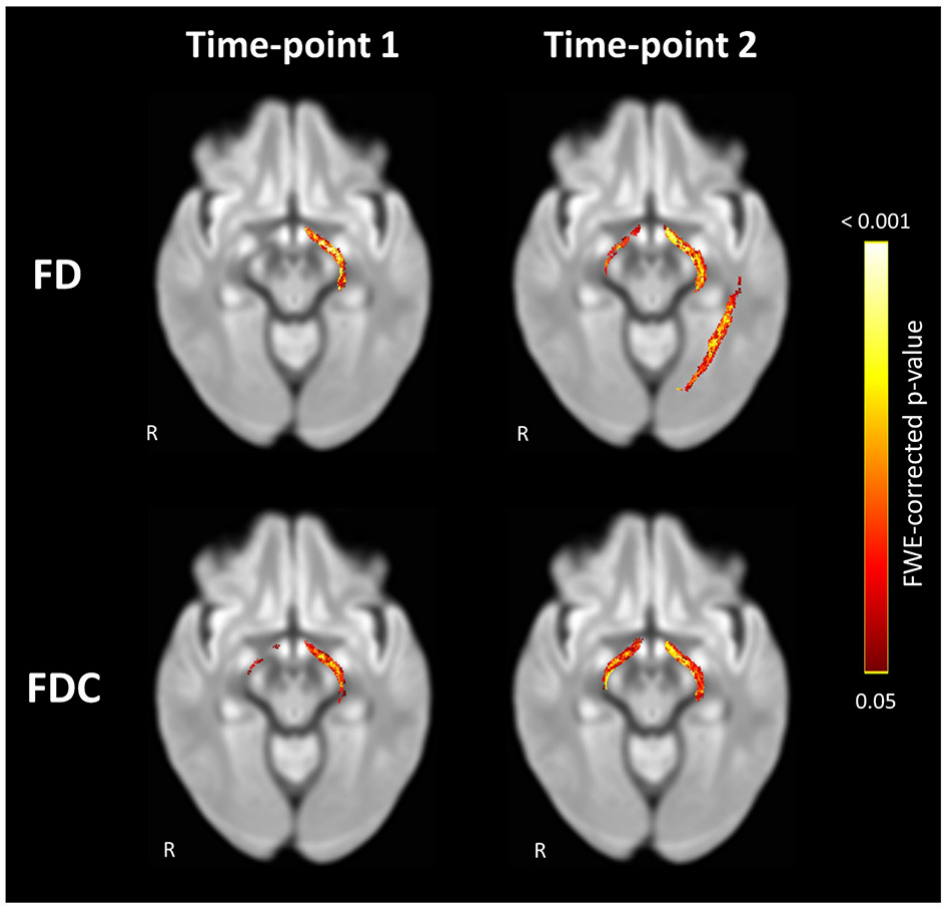
Significant loss of FD and FDC in the visual pathways of POAG patients compared to controls at Time-point 1 and Time-point 2. Loss of FD was found in the left OT at Time-point 1 and in both OTs and left OR at Time-point 2. Loss of FDC was found in both OTs at both time-points. Streamlines corresponding to fixels exhibiting significant (FWE-corrected *P* < 0.05) loss are overlaid on a representative axial slice of the inter-subject population template and colored according to their *p*-values. Images are shown in radiologic convention. FD, fiber density; FDC, fiber density and bundle cross section.

Compared to the controls, the POAG patients exhibited a significant loss of FD in the left OT at TP1 and in both OTs and left OR at TP2 (Figure 2). No significant difference in FC was found between the groups at both time-points. FDC was significantly lower in both OTs of the POAG patients at both time-points, although the right OT showed an increase in spatial pervasiveness of the FDC loss at TP2 compared to TP1 (Figure 2).

### Longitudinal Fixel-Based Analyses

**Figure 3:**
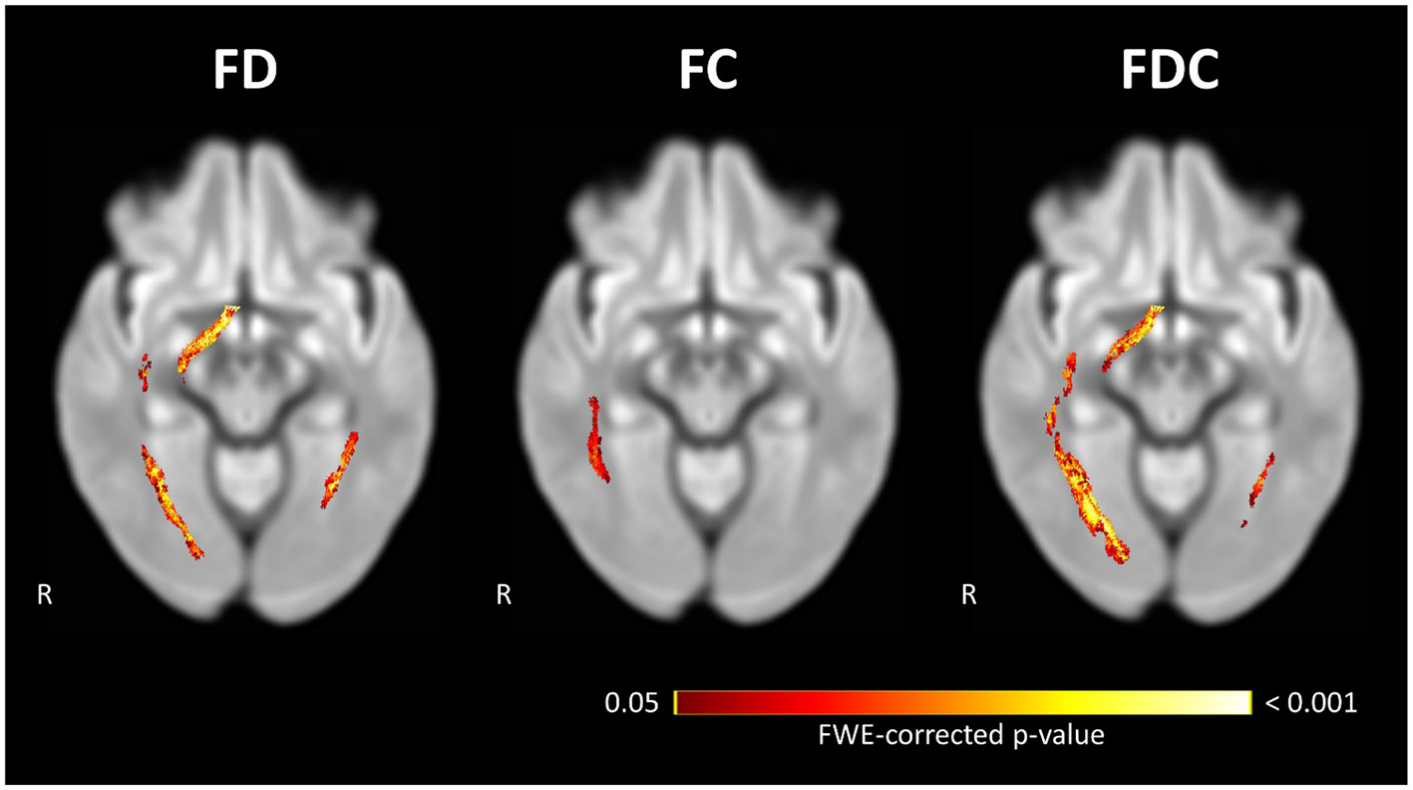
Significant loss of FD, FC, and FDC in the visual pathways of POAG patients between Time-point 1 and Time-point 2. Repeated measures analysis of POAG patients reveals a significant loss of FD and FDC at the right OT and both ORs, and a significant loss of FC in the right OR. Streamlines corresponding to fixels exhibiting significant (FWE-corrected *P* < 0.05) loss are overlaid on a representative axial slice of the inter-subject population template and colored according to their *p*-values. Images are shown in radiologic convention. FC, fiber-bundle cross section; FD, fiber density; FDC, fiber density and bundle cross section.

**Figure 4:**
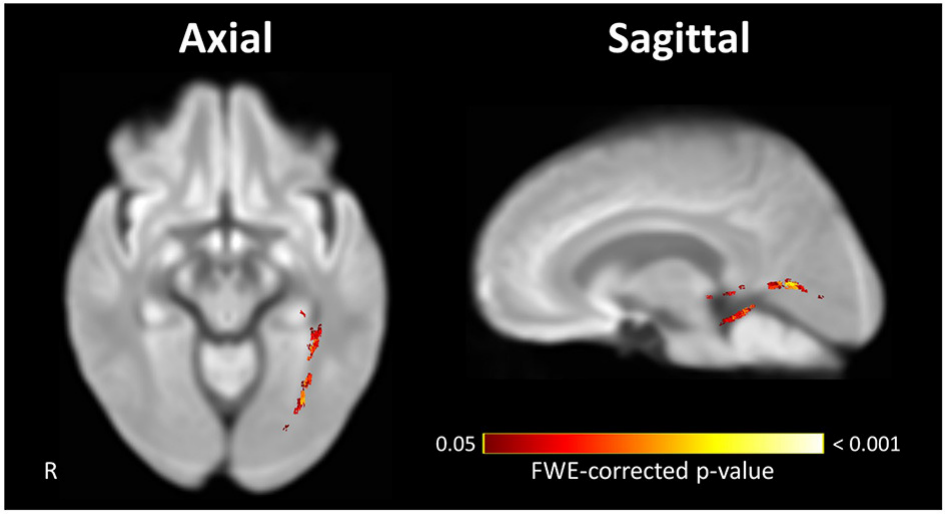
Significant difference in the rate of FD loss exhibited by visual pathways of POAG patients compared to controls. The left OR of POAG patients showed a significantly higher rate of FD loss compared to the controls. Streamlines corresponding to fixels exhibiting a significant (FWE-corrected *P* < 0.05) difference between groups are overlaid on representative axial (left) and sagittal (right) slices of the inter-subject population template and colored according to their *p*-values. Images are shown in radiologic convention. FD, fiber density.

Within-subject repeated measures analysis of POAG patients showed a significant decrease in FD and FDC in the right OT and both ORs and a decrease of FC in the right OR between the time-points (Figure 3). The left OT showed no significant change in any FBA measure between the two time-points. Following comparison of the rate of change in FBA measures in POAG patients and controls, the left OR showed a significantly higher rate of FD loss in POAG patients (Figure 4). Rate of FC and FDC changes showed no significant difference between the groups.

### Supplementary Confirmatory Analyses

In the confirmatory cross-sectional analyses, similar patterns of FBA changes were found in all repeated analyses for the time-interval-matched participants (Supplementary Figures 1, 2). While the same patterns were detected, their spatial distribution differed slightly from the results of the analyses which included all the participants. For the confirmatory longitudinal analyses, the left OR showed a significantly higher FD loss between the time-points in POAG patients (Supplementary Figure 3).

### Correlation Between FBA Measures and Clinical Tests

**Figure 5:**
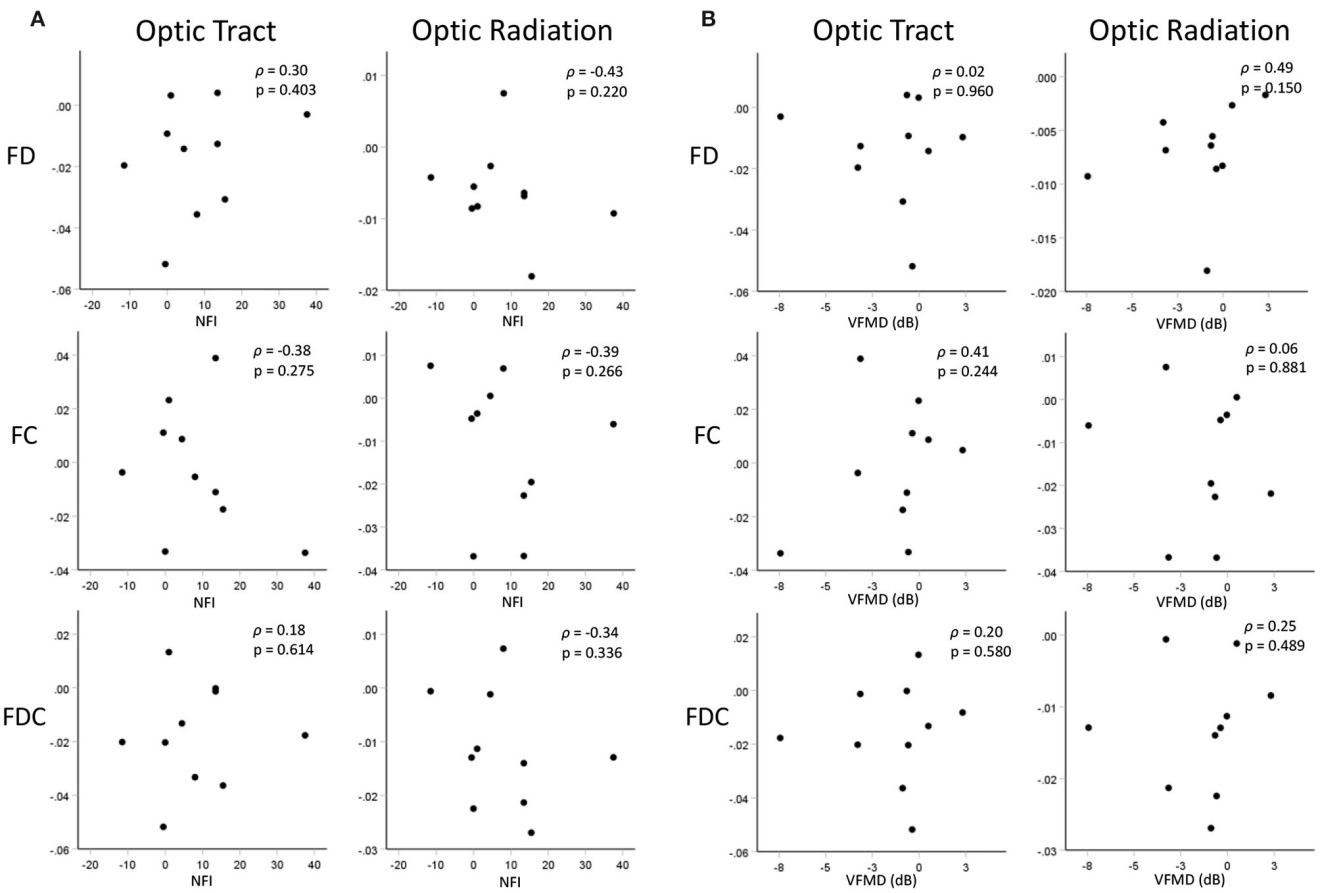
Scatterplots showing correlations between changes in FBA measures of the OTs and ORs and changes in **(A)** RNFL thickness (represented by NFI) and **(B)** visual function (represented by VDMD) over time. Displayed FBA, NFI, and VMFD values are the differences in the measures between the two time-points.

No significant correlation was found between the differences in the results of the clinical tests of glaucoma (NFI and VFMD) and the differences in FBA measures of the POAG group between the two time-points (Figure 5).

## Discussion

Our main finding is longitudinal progression of neurodegenerative WM changes in both the pre- and post-geniculate visual pathways of POAG patients. We find that WM degeneration starts at the pre-geniculate visual pathways, represented by the OTs, then spreads to the post-geniculate visual pathways, represented by the ORs. This signifies that in POAG, anterograde trans-synaptic degeneration is responsible for the spread of degeneration along the visual pathways. Furthermore, we find evidence of continued progression of post-geniculate visual pathway degeneration in the absence of detectable pre-geniculate visual pathway degenerative progression. We discuss these results, as well as their clinical implications, in more detail below.

### Evidence of Anterograde Trans-synaptic Degeneration Along the Visual Pathways in POAG

The cross-sectional analysis at TP1 revealed a loss of FD in the left OT in POAG patients, while the analysis at TP2 revealed an additional loss of FD in the right OT and the left OR. The spread of glaucomatous degeneration from OT to OR on the left side and the appearance of degeneration on the right starting at the OT and not the OR is evidence that glaucomatous degeneration in the POAG group starts at the OTs. The spread of glaucomatous degeneration from the pre-geniculate OTs to the post-geniculate ORs implicates anterograde trans-synaptic degeneration as the main mechanism of spread of glaucomatous degeneration along the visual pathways. While the ORs did not show a loss of FDC at either time-point, the spatial expansion of FDC loss over time in the right OT, in the absence of such FDC loss in the right OR, is further proof that glaucomatous degeneration starts at the pre-geniculate pathways. Moreover, the confirmatory analyses of the time-interval-matched subgroups show a similar pattern of spread, albeit on a smaller spatial scale, which can be attributed to the smaller group sizes resulting from the one-to-one matching of controls to patients. Furthermore, the repeated measures analyses of the POAG group showed a loss of FD and FDC in the right OT and both ORs over time, confirming the anterograde trans-synaptic degeneration pattern of spread. The laterality in our findings is most likely statistical in nature, and not a reflection of an underlying pathophysiological mechanism. Lateralized degeneration was reported in a previous meta-analysis of cross-sectional DWI studies of glaucoma (Li et al., 2014), although evidence of greater degeneration was found on the right rather than the left side. If the laterality was a true reflection of glaucomatous brain pathology, the side showing evidence of more advanced degeneration would have been consistent across studies.

The current findings confirm our previously proposed interpretation of the results of a cross-sectional FBA study of the visual pathways in POAG (Haykal et al., 2019). In that study, we found evidence of FD loss in both the OTs and ORs with simultaneous FC loss in the OTs only. As animal studies have shown that axonal loss precedes atrophy of WM in POAG (Ito et al., 2009), we interpreted this to be evidence of anterograde trans-synaptic degeneration. While the pattern of spread in our current study is in line with our previous FBA findings, we did not find a similar loss of FC at either time-point. A possible explanation could be that the POAG patients in the current study have not collectively reached a late enough stage of glaucomatous degeneration to exhibit detectable WM atrophy, and hence significant FC loss. Another possible explanation is that in our previous cross-sectional FBA study, we were able to use a more advanced multi-shell DWI protocol optimized specifically for FBA, while in the current retrospective longitudinal study we had to conform to the same single-shell DWI protocol used to acquire the data several years ago at TP1. Nonetheless, our present observation of progression of FD loss over time in the absence of any FC loss after accounting for age-related WM degeneration between the time-points agrees with the concept of axonal loss preceding gross WM atrophy in glaucoma.

To our knowledge, all previous studies of WM degeneration in POAG have been cross-sectional in nature, making direct comparison to our current work difficult. Some cross-sectional whole-brain studies of POAG have found evidence of degenerative changes outside the visual system (Frezzotti et al., 2014, 2016), suggesting that POAG is a global neurodegenerative disease of the brain and that glaucomatous changes found at the level of the eye are secondary to this global degeneration. These findings imply that retrograde trans-synaptic degeneration is responsible for the spread of glaucomatous degeneration along the visual pathways, which would contradict our current findings. However, we note that to obtain their results, such studies had to rely on lenient statistical methods, which calls into question the reliability of their findings. For example, Frezzotti et al. (2014, 2016) performed tract-based spatial statistics (TBSS) without correction for multiple comparisons. Similar TBSS studies of POAG that applied FWE-correction did not find evidence of WM degeneration outside the visual pathways (Chen et al., 2013; Lu et al., 2013). Furthermore, as mentioned, all current evidence of retrograde trans-synaptic degeneration has been derived from cross-sectional investigations, which are not suitable for studying the progression of degenerative changes over time.

### Progression of Post-geniculate Visual Pathway Degeneration in the Absence of Detectable Pre-geniculate Visual Pathway Degeneration

While only the repeated measures analyses show a loss of FD in the right OT and OR, both longitudinal analyses demonstrate FD loss between the time-points in the left OR with no detectable FD loss in the left OT. This cannot be attributed to an overall absence of FD loss in the left OT, as the cross-sectional analyses at TP1 and TP2 show a loss of FD in the left OT in comparison to the controls. Therefore, while the left OT exhibits signs of glaucomatous degeneration, there is no evidence that this degeneration progressed significantly between the time-points. The absence of a detectable degenerative progression in the pre-geniculate OT coupled with the presence of a degenerative progression in the post-geniculate OR suggests that trans-synaptic spread of glaucomatous degeneration is a prolonged process that does not necessarily cease once pre-synaptic degeneration plateaus have been reached.

A similar pattern of continued trans-synaptic degenerative spread following the arrest of degeneration of the source has been previously described in the visual pathways in both antero- and retrograde directions. A longitudinal study of multiple sclerosis patients who suffered their first episodes of optic neuritis found evidence of continued anterograde trans-synaptic degeneration affecting their ORs for up to at least a year following the episodes, an observation the investigators referred to as a “trans-synaptic lag effect” (Tur et al., 2016). Another longitudinal study of patients suffering from homonymous visual field defects due to post-geniculate injury found evidence of continued retrograde trans-synaptic degeneration in the optic nerves for up to 10 years in some of the patients (Mitchell et al., 2015). Therefore, our study supports the notion that prolonged trans-synaptic spread is a common characteristic of trans-synaptic degeneration along the visual pathways. Importantly, this observation has not been documented in POAG before, and can potentially change the way central degeneration in POAG is viewed and clinically managed.

### Correlation Between FBA Measures and Clinical Tests of Glaucoma

In the current study, we found no statistically significant correlation between the changes in structural and functional clinical tests of glaucoma and the changes in FBA measures in POAG patients. This lack of correlation could be attributed to our relatively moderate sample sizes, in addition to some missing data points at TP1 (Table 1; Supplementary Table 1). Additionally, the RNFL thickness was assessed using laser polarimetry, which was found to be less sensitive to retinal changes over time in comparison to other methods such as optical coherence tomography (Nomoto et al., 2014). Furthermore, DWI data was acquired using a single-shell scanning protocol which was not optimized for FBA. Most likely, the lack of correlation found in the current study is the combined outcome of all the suggested causes.

### Clinical Implications

Characterizing the pattern of spread of glaucomatous degeneration along the visual pathways contributes to our understanding of the underlying pathophysiology of POAG. This knowledge could have great significance to POAG diagnostics and therapeutics.

Our current findings could play a role in the development of novel glaucoma therapies. For example, novel neuroprotective therapies aiming to stop the progression of glaucoma would benefit from being able to assess glaucomatous spread along the visual pathways, which can only be determined once the pattern of this spread has been identified. Other novel therapies such as RGC transplantation could also benefit from this knowledge, as transplanting RGCs to the pre-geniculate pathway would be ineffective if glaucomatous degeneration was found to start at the post-geniculate pathway.

Identifying the previously mentioned “trans-synaptic lag effect” in POAG could also prove to be beneficial to glaucoma treatment in the future. By further studying this effect and identifying its time frame more precisely, viable post-geniculate neurons corresponding to degenerated pre-geniculate neurons could be salvaged before they succumb to trans-synaptic degeneration A similar argument related to retrograde trans-synaptic degeneration following injury to the post-geniculate pathways has been presented by de Vries-Knoppert and colleagues (de Vries-Knoppert et al., 2019). Such preventative measures could become critical in the context of the development of RGC transplantation efforts.

### Limitations and Future Directions

Our current study has several limitations. The relatively small pool of potential participants for this follow-up study, in addition to being restricted by the number of participants who agreed to return and who were also eligible for recruitment, limited our present sample sizes. Nevertheless, our sample sizes are still in line with a number of previous cross-sectional studies of WM degeneration in POAG (Frezzotti et al., 2014; Kaushik et al., 2014; Zhou et al., 2017; Haykal et al., 2019). Another limitation is the different time intervals experienced by the participants of the two previous studies, and an overall time interval difference between the included POAG patients and the controls. To address this issue, we performed a time-interval-matched analysis, the results of which confirmed our main findings. Future studies investigating glaucomatous spread could consider increasing the number of follow-up time-points, in order to increase the precision with which the time frames of degeneration and the trans-synaptic lag effect can be studied. Such future prospectively planned longitudinal studies could further benefit from more consistent time intervals, the inclusion of early stage POAG patients, larger sample sizes and utilizing a DWI acquisition protocol optimized for higher-order diffusion models. Moreover, including participants who are still in a “glaucoma suspect” stage would be ideal. However, to retain meaningful numbers of participants that have progressed into more advanced stages of glaucoma would require huge numbers of initial inclusions, which would render such a study prohibitively expensive.

## Conclusions

We find that the degenerative changes present throughout the visual pathways of POAG patients are most likely the result of anterograde trans-synaptic spread of glaucomatous degeneration originating at the pre-geniculate pathways. Furthermore, trans-synaptic spread of glaucomatous degeneration is a prolonged process which continues in the absence of detectable pre-geniculate visual pathway degeneration. This suggests the presence of a time window for salvaging intact post-geniculate pathways, which could prove to be a viable therapeutic target in the future.

## Data Availability Statement

The raw data supporting the conclusions of this article will be made available by the authors, without undue reservation.

## Ethics Statement

The studies involving human participants were reviewed and approved by the ethics board of the University Medical Center Groningen. The patients/participants provided their written informed consent to participate in this study.

## Author Contributions

SH: conceptualization, methodology, validation, formal analysis, investigation, data curation, writing - original draft, and visualization. NJ: conceptualization, resources, writing - review and editing, supervision, and funding acquisition. FC: conceptualization, resources, writing - review and editing, supervision, project administration, and funding acquisition. All authors contributed to the article and approved the submitted version.

## Conflict of Interest

The authors declare that the research was conducted in the absence of any commercial or financial relationships that could be construed as a potential conflict of interest.
