# Supplementary material for: Progression of Visual Pathway Degeneration in Primary Open-Angle Glaucoma: A Longitudinal Study
Source: Front Hum Neurosci. 2021 Mar 29;15:630898. doi: 10.3389/fnhum.2021.630898 (PMC8039117; doi:10.3389/fnhum.2021.630898)
Supplement: Supplementary file 1 [file Image_1.PDF]

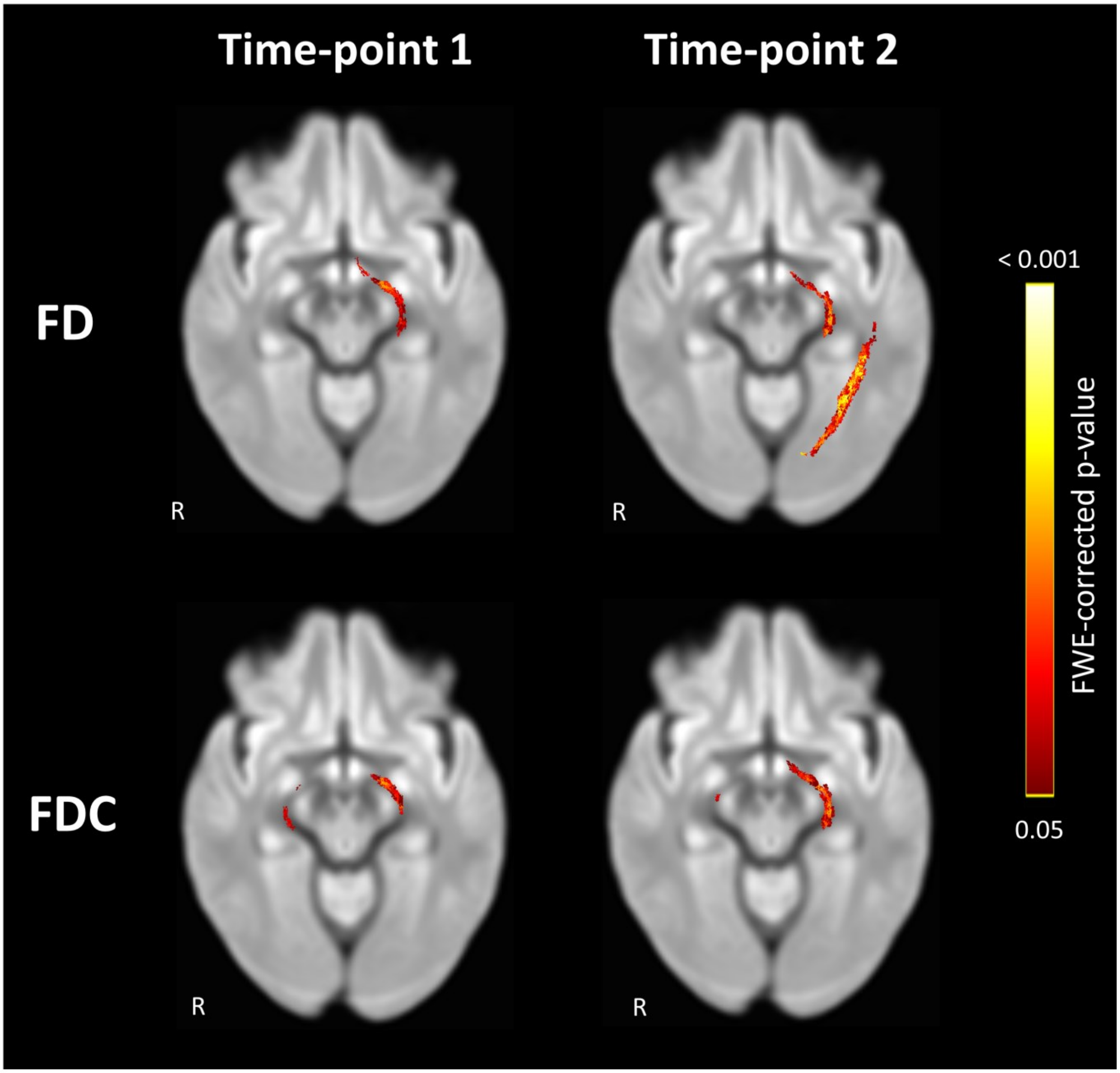

Supplementary Figure S1. Significant loss of FD and FDC in the visual pathways of POAG patients compared to time-interval-matched controls at Time-point 1 and Time-point 2. Loss of FD was found in the left OT at time-point 1 and in left OT and OR at time-point 2. Loss of FDC was found in both OTs at both time-points. Streamlines corresponding to fixels exhibiting significant (FWE-corrected P < 0.05) loss are overlaid on a representative axial slice of the inter-subject population template and colored according to their p-values. Images are shown in radiologic convention. FD: fiber density; FDC: fiber density and bundle cross section.
