# Supplementary material for: Progression of Visual Pathway Degeneration in Primary Open-Angle Glaucoma: A Longitudinal Study
Source: Front Hum Neurosci. 2021 Mar 29;15:630898. doi: 10.3389/fnhum.2021.630898 (PMC8039117; doi:10.3389/fnhum.2021.630898)
Supplement: Supplementary file 2 [file Image_2.PDF]

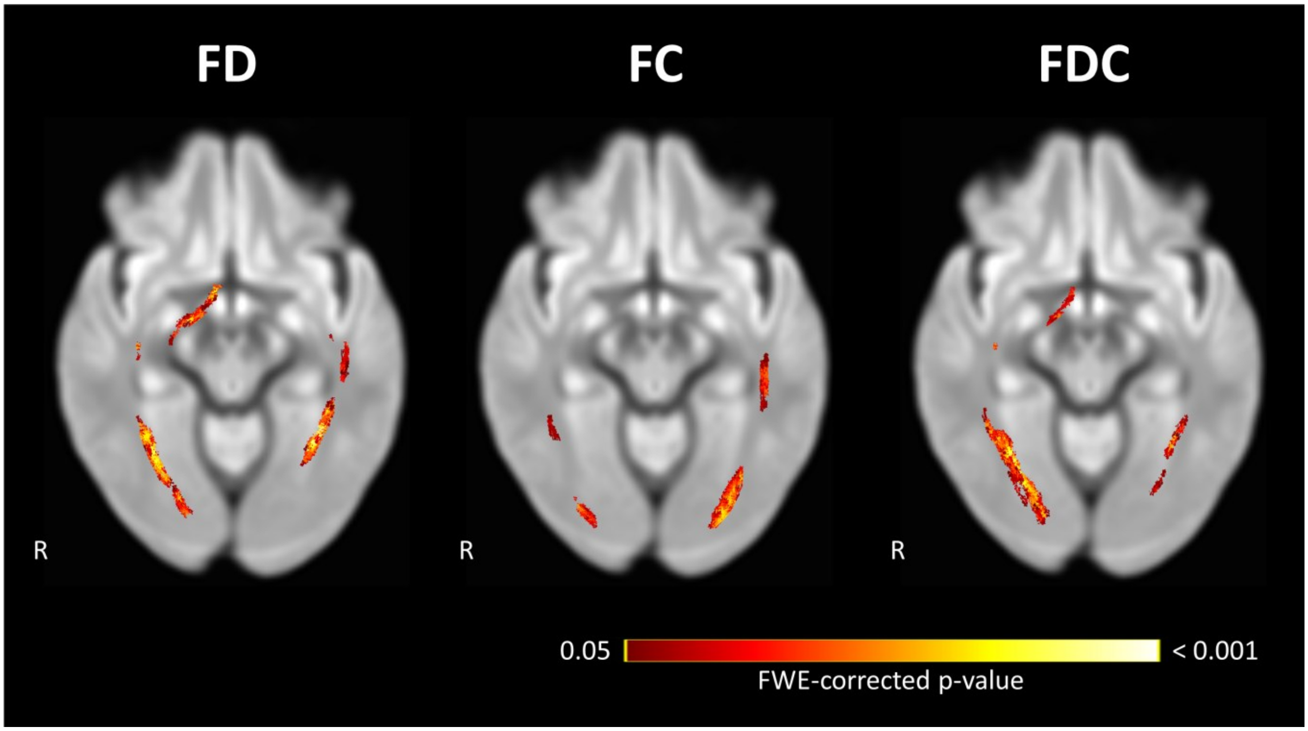

Supplementary Figure S2. Significant loss of FD, FC and FDC in the visual pathways of time-interval-matched POAG patients between Time-point 1 and Time-point 2. Repeated measures analysis of time-interval-matched POAG patients reveal a significant loss of FD and FDC at the right OT and both ORs, and a significant loss of FC in both ORs. Streamlines corresponding to fixels exhibiting significant (FWE-corrected $P < 0.05$) loss are overlaid on a representative axial slice of the inter-subject population template and colored according to their p-values. Images are shown in radiologic convention. Fiber-bundle cross section (FC), FD: fiber density; FDC: fiber density and bundle cross section.
