# Supplementary material for: Progression of Visual Pathway Degeneration in Primary Open-Angle Glaucoma: A Longitudinal Study
Source: Front Hum Neurosci. 2021 Mar 29;15:630898. doi: 10.3389/fnhum.2021.630898 (PMC8039117; doi:10.3389/fnhum.2021.630898)
Supplement: Supplementary file 3 [file Image_3.PDF]

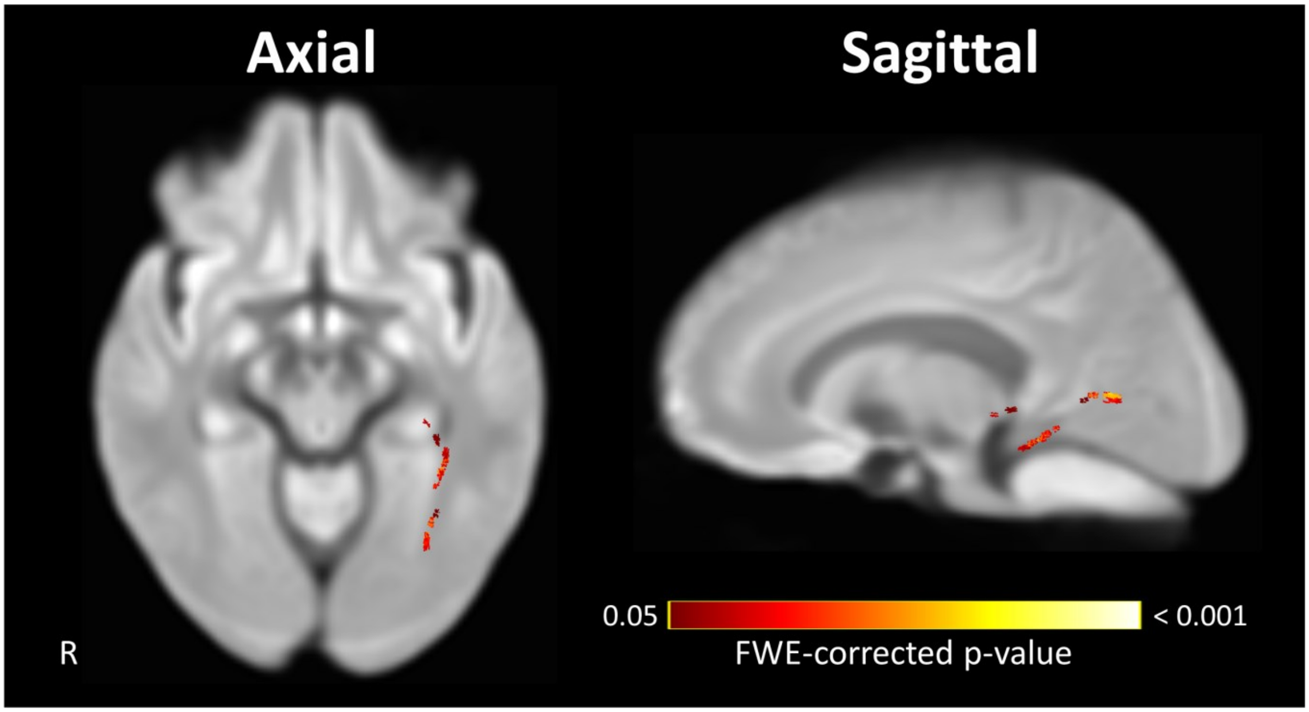

Supplementary Figure S3. Significant difference in FD loss exhibited by visual pathways of POAG patients compared to controls. The left OR of POAG patients showed significantly higher FD loss compared to the controls. Streamlines corresponding to fixels exhibiting a significant (FWE-corrected $P < 0.05$) difference between groups are overlaid on representative axial (left) and sagittal (right) slices of the inter-subject population template and colored according to their p-values. Images are shown in radiologic convention.
