# Supplementary material for: Progression of Visual Pathway Degeneration in Primary Open-Angle Glaucoma: A Longitudinal Study
Source: Front Hum Neurosci. 2021 Mar 29;15:630898. doi: 10.3389/fnhum.2021.630898 (PMC8039117; doi:10.3389/fnhum.2021.630898)
Supplement: Supplementary file 4 [file Table_1.DOCX]

**Supplementary Table S1. ﻿**Glaucoma duration and outcome of ophthalmic tests in POAG Group

|  | **Glaucoma** | | **Pre-** |  | **IOP** | | | | |  | **NFI** | | | | |  | **VFMD (dB)** | | | | |
| --- | --- | --- | --- | --- | --- | --- | --- | --- | --- | --- | --- | --- | --- | --- | --- | --- | --- | --- | --- | --- | --- |
|  | **duration (y)** | | **treatment** |  |  |  |  |  |  |  |  |  |  |  |  |  |  |  |  |  |  |
|  |  |  | **IOP*** |  | **TP1** | |  | **TP2** | |  | **TP1** | |  | **TP2** | |  | **TP1** | |  | **TP2** | |
|  |  |  |  |  |  |  |  |  |  |  |  |  |  |  |  |  |  |  |  |  |  |
| **Patient** | **TP1** | **TP2** |  |  | **OD** | **OS** |  | **OD** | **OS** |  | **OD** | **OS** |  | **OD** | **OS** |  | **OD** | **OS** |  | **OD** | **OS** |
| **no.** |  |  |  |  |  |  |  |  |  |  |  |  |  |  |  |  |  |  |  |  |  |
|  |  |  |  |  |  |  |  |  |  |  |  |  |  |  |  |  |  |  |  |  |  |
| 1 | 0 | 9 | 24 |  | 19 | 20 |  | 11.7 | 10 |  | 80 | 30 |  | 81 | 28 |  | -2.70 | -2.20 |  | -3.40 | -2.23 |
| 2 | 9 | 19 | 38 |  | 23 | 22 |  | 14 | 14.7 |  | 48 | 36 |  | 74 | 41 |  | -1.40 | -0.20 |  | -2.25 | -1.10 |
| 3 | 10 | 14 | 30 |  | 16 | 17 |  | 16 | 16 |  | 32 | 88 |  | 59 | 88 |  | -1.43 | -11.81 |  | -4.24 | -15.29 |
| 4 | 7 | 12 | 26 |  | 20 | 15 |  | 19 | 9.7 |  | 23 | 49 |  | 43 | 56 |  | -2.06 | -12.73 |  | -3.52 | -12.6 |
| 5 | 5 | 9 | 33 |  | 8 | 5 |  | 12 | 8 |  | - | - |  | 61 | 57 |  | -10.77 | -4.81 |  | -6.36 | -4.56 |
| 6 | 4 | 9 | 26 |  | 11 | 11 |  | 9.3 | 9.7 |  | 93 | 29 |  | 71 | 28 |  | -6.56 | -0.24 |  | -12.87 | -0.50 |
| 7 | 3 | 11 | 26 |  | - | - |  | 17.3 | 25.3 |  | - | - |  | 19 | 69 |  | - | - |  | 0.52 | -4.32 |
| 8 | 11 | 15 | 56 |  | 18 | 16 |  | 14.7 | 16 |  | 23 | 49 |  | 34 | 40 |  | -1.36 | -29.05 |  | -2.36 | -28.11 |
| 9 | 4 | 9 | 18 |  | 14 | 14 |  | 11 | 14 |  | 50 | 16 |  | 50 | 16 |  | -11.50 | 0.46 |  | -11.76 | -0.44 |
| 10 | 11 | 20 | 27 |  | 14 | 14 |  | 9 | 9.7 |  | 71 | 18 |  | 73 | 32 |  | - | - |  | -29.58 | -1.91 |
| 11 | 2 | 11 | 39 |  | - | - |  | 12 | 11 |  | 55 | 54 |  | 91 | 93 |  | - | - |  | -18.57 | -23.1 |
| 12 | 7 | 11 | 30 |  | 12 | 13 |  | 9 | 11.3 |  | 30 | 98 |  | 39 | 98 |  | -2.06 | -23.75 |  | -0.92 | -23.9 |
|  |  |  |  |  |  |  |  |  |  |  |  |  |  |  |  |  |  |  |  |  |  |

IOP = intraocular pressure; NFI = nerve fiber indicator; OD = right eye; OS = left eye; VFMD = visual field mean deviation.

*Reported values are of the eye with higher pre-treatment IOP
