# Supplementary material for: Progression of Visual Pathway Degeneration in Primary Open-Angle Glaucoma: A Longitudinal Study
Source: Front Hum Neurosci. 2021 Mar 29;15:630898. doi: 10.3389/fnhum.2021.630898 (PMC8039117; doi:10.3389/fnhum.2021.630898)
Supplement: Supplementary file 5 [file Table_2.DOCX]

**Supplementary Table S2.** Time-interval-matched POAG patients and controls

|  | POAG | | |  | Time-interval-matched controls | | |
| --- | --- | --- | --- | --- | --- | --- | --- |
| Patient No. | Age at TP1 (y) | Age at TP2 (y) | Time interval (y) |  | Age at TP1 (y) | Age at TP2 (y) | Time interval (y) |
|  |  |  |  |  |  |  |  |
| 2 | 60 | 69 | 9.5 |  | 60 | 69 | 9.25 |
| 3 | 73 | 77 | 4.42 |  | 81 | 85 | 4.5 |
| 4 | 72 | 76 | 4.5 |  | 66 | 70 | 4.5 |
| 5 | 57 | 61 | 4.17 |  | 55 | 59 | 4 |
| 6 | 68 | 72 | 4.5 |  | 68 | 72 | 4.42 |
| 7 | 53 | 61 | 7.83 |  | 50 | 58 | 7.83 |
| 8 | 55 | 58 | 3.67 |  | 67 | 70 | 3.5 |
| 9 | 54 | 59 | 4.5 |  | 53 | 58 | 4.5 |
| 12 | 54 | 57 | 3.83 |  | 55 | 59 | 4 |
|  |  |  |  |  |  |  |  |
| **Mean (SD)** | 61.7 (9.8) | 66.7 (9.1) | 5.2 (2.0) |  | 60.7 (8.1) | 65.6 (8.0) | 5.2 (2.0) |
|  |  |  |  |  |  |  |  |
